# Human malignant mesothelioma is recapitulated in immunocompetent BALB/c mice injected with murine AB cells

**DOI:** 10.1038/srep22850

**Published:** 2016-03-10

**Authors:** Rosanna Mezzapelle, Eltjona Rrapaj, Elena Gatti, Chiara Ceriotti, Francesco De Marchis, Alessandro Preti, Antonello E. Spinelli, Laura Perani, Massimo Venturini, Silvia Valtorta, Rosa Maria Moresco, Lorenza Pecciarini, Claudio Doglioni, Michela Frenquelli, Luca Crippa, Camilla Recordati, Eugenio Scanziani, Hilda de Vries, Anton Berns, Roberta Frapolli, Renzo Boldorini, Maurizio D’Incalci, Marco E. Bianchi, Massimo P. Crippa

**Affiliations:** 1 Chromatin Dynamics Unit, Division of Genetics and Cell Biology, San Raffaele Hospital, Milano, Italy; 2 HMGBiotech, Milano, Italy; 3 Experimental Imaging Center, San Raffaele Hospital, Milano, Italy; 4 Medical Physics Unit, San Raffaele Hospital, Milano, Italy; 5 Diagnostic Radiology Unit, San Raffaele Hospital, Milano, Italy; 6 IBFM-CNR, Segrate, Italy; 7 Health Sciences Dept., Milano Bicocca University, Milano, Italy; 8 Pathological Anatomy Laboratory, San Raffaele Hospital, Milano, Italy; 9 Molecular Oncology Unit, San Raffaele Hospital, Milano, Italy; 10 ISTOVET, Besana in Brianza, Monza e Brianza, Italy;; 11 Fondazione Filarete, Milano, Italy; 12 Università degli Studi, Milano, Italy; 13 Division of Molecular Genetics, The Netherlands Cancer Institute, Amsterdam, The Netherlands; 14 Mario Negri Institute, Milano, Italy; 15 Division of Pathology “Maggiore Della Carità” Hospital, Novara, Italy; 16 San Raffaele Vita-Salute University, Milano, Italy

## Abstract

Malignant Mesothelioma is a highly aggressive cancer, which is difficult to diagnose and treat. Here we describe the molecular, cellular and morphological characterization of a syngeneic system consisting of murine AB1, AB12 and AB22 mesothelioma cells injected in immunocompetent BALB/c mice, which allows the study of the interplay of tumor cells with the immune system. Murine mesothelioma cells, like human ones, respond to exogenous High Mobility Group Box 1 protein, a Damage-Associated Molecular Pattern that acts as a chemoattractant for leukocytes and as a proinflammatory mediator. The tumors derived from AB cells are morphologically and histologically similar to human MM tumors, and respond to treatments used for MM patients. Our system largely recapitulates human mesothelioma, and we advocate its use for the study of MM development and treatment.

Malignant mesothelioma (MM) is an aggressive tumor arising from the cells lining the pleural, peritoneal and pericardial cavity, and exposure to asbestos is the major risk factor^1^. Inhaled asbestos fibers cannot be eliminated and generate a chronic inflammatory milieu, which is conducive to tumor development. In addition, individuals carrying mutations in the *BAP1* gene are at higher risk of developing MM^2^,^3^,^4^,^5^. Prognosis for this cancer is poor because of late-stage diagnosis and resistance to current conventional therapies^6^,^7^. Guidelines for the diagnosis of MM have been recently published^3^ that recommend the simultaneous use of several parameters. However, diagnoses are still largely based on immunohistochemical markers^8^. The gold standard in treatment is currently cisplatin (DDP) and pemetrexed^9^. However, patient survival is extended, on average, only 12 months; thus there is an urgent need for more effective treatments. Preclinical studies on MM rely mostly on xenotransplants of human mesothelioma cell lines into the peritoneum of SCID mice (see for instance ref. 10), but have the major limitation that the interplay between the tumor and lymphocytes cannot be studied in immunocompromised mice. Notably, High Mobility Group Box 1 protein (HMGB1) is a key player both in the ethiogenesis of MM^11^ and in eliciting innate and adaptive immune responses, including immunogenic cell death (ICD). It is therefore important to validate immunocompetent animal models of MM.

HMGB1 is a Damage Associated Molecular Pattern and alerts the immune system to cell death^12^. HMGB1 is passively released by primary human mesothelial cells exposed to asbestos, recruits macrophages and thus contributes to the initial stages of inflammation, inducing the secretion of TNF-α and other cytokines^11^. When mesothelial cells become transformed in an HMGB1-rich environment, most of the resulting MM cells require HMGB1 to grow and to invade nearby tissues; accordingly, abrogation of HMGB1 function may have therapeutic efficacy^10^.

Immunogenic cell death is a form of apoptosis caused by specific antitumor compounds, such as anthracyclines, oxaliplatin and bortezomib, or radiotherapy, that can induce an effective antitumour immune response through activation of specific T cell responses. It is thus functionally very different from “normal” apoptosis, which is non-immunogenic or even tolerogenic^13^. ICD has three major hallmarks: the release of ATP into the extracellular space, the exposure of calreticulin on the cell surface, and the release of HMGB1. Whereas the induction of ICD can be therapeutically advantageous, this can only be studied in immunocompetent mice.

To set up a model where the interplay between MM and the immune system can be investigated, we focused our attention on murine mesothelioma cell lines (AB1, AB12 and AB22) that were derived from spontaneously arising mesotheliomas in BALB/c mice injected intraperitoneally with asbestos^14^. These cells are routinely used as model systems for *in vitro* and *in vivo* studies^4^,^15^,^16^,^17^,^18^,^19^,^20^,^21^,^22^. However, the characterization of these cells and of the tumors that develop following their intraperitoneal transplantation was mainly based on causative agent, morphology and ultrastructure^14^.

Here we describe a phenotypical and molecular characterization of AB cell lines and of the tumor masses they produce. In particular, we have explored their genetic setup, characterized their markers, and their response to HMGB1. We employed multiple imaging techniques to study the growth and vascularization of tumor masses generated by the intraperitoneal injection of AB cells in BALB/c mice. We provide evidence that tumors obtained by injecting AB cells in immunocompetent mice are considerably similar to human malignant mesotheliomas. The mouse model appears to recapitulate the early stages of mesothelioma development, which is useful to identify early biomarkers. Moreover, murine MM masses respond to treatment with chemotherapeutics routinely used to treat mesothelioma patients. Our results support the use of this mouse MM model in preclinical studies.

## Results and Discussion

### Characterization of murine MM cell lines

**Figure 1:**
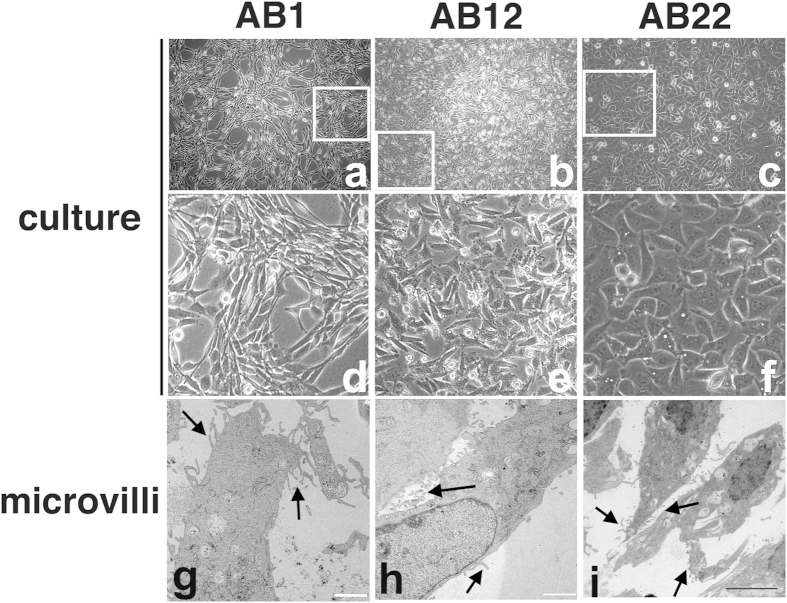
Morphology of murine MM cell lines. Cultured AB1, AB12 and AB22 cells, showing morphological features corresponding to sarcomatoid (AB1; (**a,d**)), biphasic (AB12; (**b,e**)) and stellate/epithelioid (AB22; (**c,f**)) phenotypes. Boxed areas in a, b and c are enlarged in (**d**–**f**), respectively, to better appreciate cell morphology. Transmission electron microscopy reveals the presence of microvilli (**g–i**), a hallmark of mesothelial cells; Bars in g and h = 2 μm; bar in i = 4 μm.

AB1, AB12 and AB22 murine MM cell lines were derived from female BALB/c mice injected i.p. with asbestos fibers^14^ and have similar phenotypes to the sarcomatoid, biphasic and epithelioid cells of human mesothelioma, respectively (Fig. 1a–f). On the surface of such cells we detected microvilli (Fig. 1g–i), an important hallmark of mesothelial cells^23^.

The original cell lines were: 1) re-injected in male BALB/c mice and re-isolated (and named AB1-B/c; AB12-B/c; AB22-B/c); 2) infected with a lentiviral vector constitutively expressing the luciferase gene (and named AB1-LUC; AB12-LUC; AB22-LUC); or 3) underwent both manipulations sequentially (and named AB1-B/c-LUC; AB12-B/c-LUC; AB22-B/c-LUC). The manipulated cell lines displayed the same features of the original strains (see below) and were interchangeably used in the experiments.

**Figure 2:**
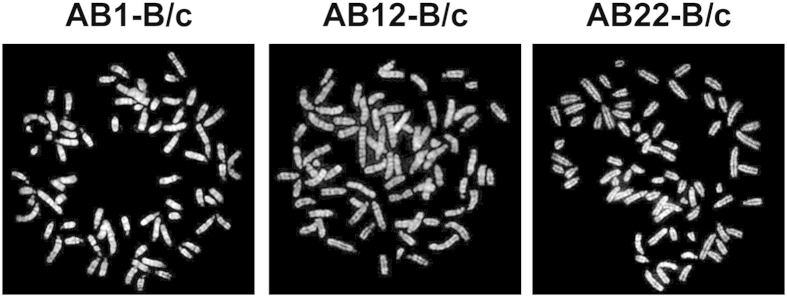
Karyotype of murine MM cells. Representative metaphases were visualized as described in Materials and Methods; 20 metaphases for each cell line were counted.

In line with what reported for human MM cell lines^23^,^24^,^25^,^26^,^27^, murine AB lines have a hypervariable modal chromosome number, chromosomal heterogeneity and polyploidy (Fig. 2a–c and Supplementary Figure 1a). AB1-B/c and AB22-B/c cells are hyper-triploid (61–76 chromosomes and 64–75 chromosomes, respectively, with two nuclei of AB1-B/c cells containing 131 and 133 chromosomes), while AB12-B/c cells are nearly tetraploid (73–80 chromosomes). Genome sequencing of the three original cell lines, albeit at low coverage (42, 41.5 and 40.7 M reads for AB1, AB12 and AB22 cells respectively), show partial chromosomal imbalances (duplications or deletions) with no gain or loss of entire chromosomes (Supplementary Figure 1).

**Figure 3:**
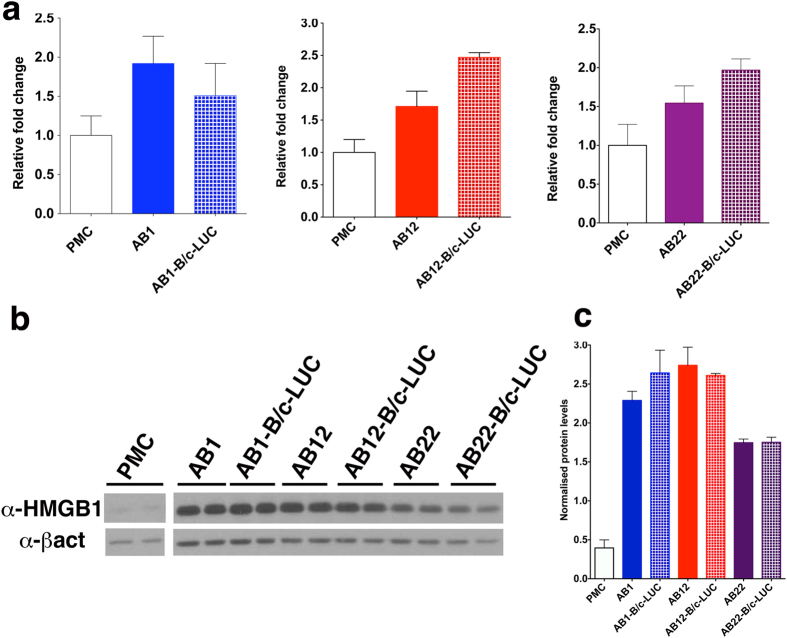
Murine MM cells express high levels of HMGB1 mRNA and secrete the protein. (**a**) Total RNA from primary mesothelial cells was retrotranscribed and quantified by qPCR, as described in Materials and Methods. HMGB1 mRNA levels are about twofold higher in MM cell lines than in primary mesothelial cells (PMC). The bars represent standard deviation (n = 3). (**b**) Equal aliquots from total lysates of primary mesothelial cells or of the indicated cell lines were run (in duplicate) on SDS-PAGE, and western blotted with anti-HMGB1 and anti-β-actin antibodies. The filters were exposed for the same time showing that HMGB1 signal from primary mesothelial cells is barely detectable. (**c**) Quantification of HMGB1 bands in (**b**), normalized to β-actin levels. The bars represent standard deviation (n = 2).

HMGB1 has been shown to be crucial for MM development^10^,^11^. Endogenous levels of HMGB1 mRNA are approximately twofold higher in AB cell lines than in primary mesothelial cells (Fig. 3a and Supplementary Figure 2). Likewise, western blot analysis shows that the levels of endogenous HMGB1 protein are higher in AB cells than in primary mesothelial cells (Fig. 3b,c). In the latter, HMGB1 could only be observed by overexposure of the filter.

**Figure 4:**
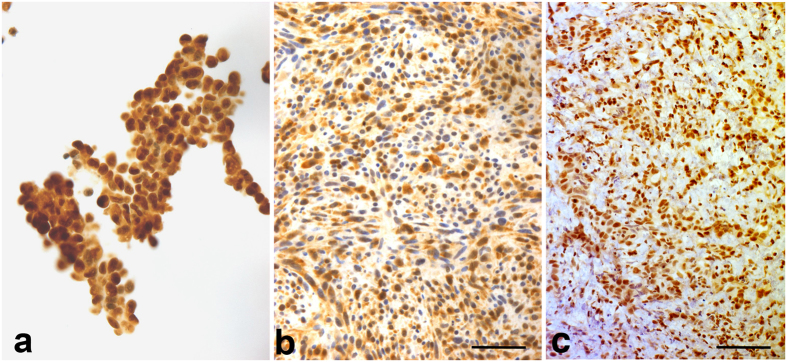
Murine MM cells and tumors show both nuclear and cytoplasmic localization of HMGB1. Nuclear and cytoplasmic localization of HMGB1 in cultured cells and tumor tissues. A polyclonal anti-HMGB1 antibody was used to immunostain (**a**) a cytopellet of cultured AB1-B/c-LUC cells, (**b**) a section of a tumor derived from them and (**c**) a section of a human MM. Bars = 100 μm.

**Figure 5:**
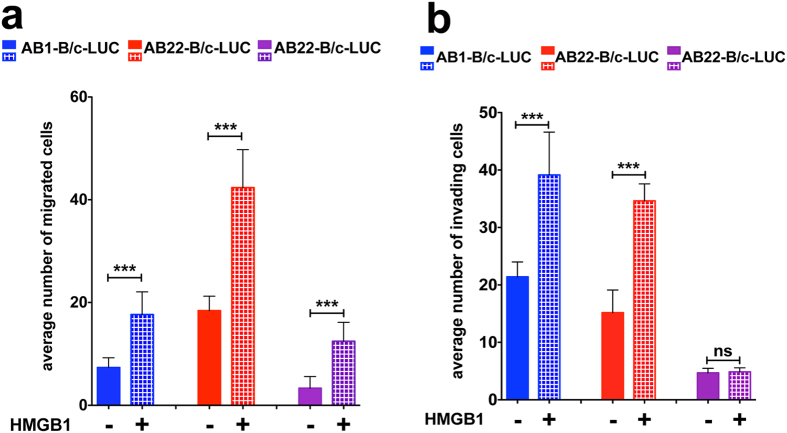
AB cells respond to extracellular HMGB1. (**a**) HMGB1 (30 ng/ml) acts as a chemoattractant for AB cell lines in Boyden chamber assays. The bars represent standard deviation (n = 3). (**b**) HMGB1 (30 ng/ml) increases the invasive potential of AB1-B/c-LUC and AB12-B/c-LUC cells, but not of AB22-B/c-LUC cells, in Boyden chamber invasion assays. The bars represent standard deviation (n = 3); p < 0.0001 (***). All experiments were repeated at least twice with similar results.

Immunostaining of cultured murine MM cells and tumors derived from them showed that HMGB1 is localized in the nucleus, but also in the in the cytoplasm, as expected if the protein is actively secreted (Fig. 4a–c and Supplementary Figure 3). In agreement with this, ELISA assays detect 20–30 fold higher levels of secreted HMGB1 in the culture medium of murine MM cells compared to that of primary mesothelial cells (Table 1). Furthermore, HMGB1 chemoattracts all AB cell lines and promotes invasion of AB1- and AB12-B/c-LUC cells, but not of AB22-B/c-LUC cells (Fig. 5a,b).

Thus, murine MM cell lines recapitulate the features of human MM cell lines, including their response to HMGB1, supporting their migration and invasion^10^.

### Characterization of tumor masses generated by murine MM cell lines

**Figure 6:**
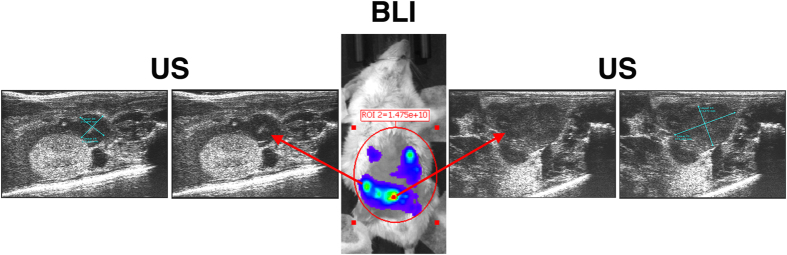
Tumor detection *in vivo* by BLI and US imaging. 15–20 days following injection of 7 × 10^4^ AB1-B/c-LUC cells, mice developed tumor masses that were detected by BLI and US. The mass identified by US and shown in the panels on the right was estimated to measure 3.5 × 5.4 mm and yielded a higher BLI signal, whereas the one shown in the panels on the left was estimated to measure 2.5 × 2.5 mm and had a lower BLI signal. In both cases the BLI signal is sufficiently strong, allowing their detection as individual masses.

I.p. injection of AB cells, whether manipulated or not, in BALB/c mice yield sizable tumor masses in 2–3 weeks. At early stages of tumor development (0–12 days) their growth was followed by the increase in BLI signal only. At these stages tumor masses and, for instance, lymph nodes have similar sizes and ultrasound (US) scans cannot tell them apart. However, differences between strong vs. weak BLI signals of larger tumor masses can be clarified by coupling BLI with US scans, which provide information on tumor size and spatial (abdominal) location, and reveal their relationship with other organs of the abdominal cavity (Fig. 6).

**Figure 7:**
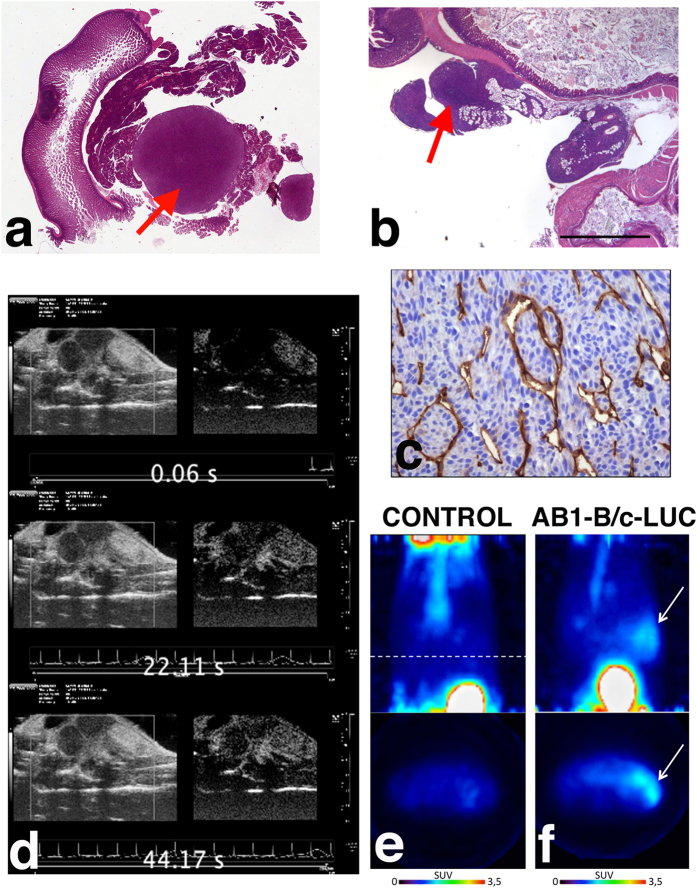
Vascularization of murine MM tumors. Sarcomatoid tumors (red arrows) generated by injection of AB1 cells in BALB/c mice were explanted and stained with H&E (**a,b**); bar, 2 mm. No necrosis is detectable in the tumor masses. Immunostaining with anti-CD31 antibodies of sections from the tumor in (**c**) reveals several small vessels in the inner mass. (**d**) A movie montage (from Supplementary Movie 1) shows that the intravenously delivered contrast bolus infiltrates the tumor masses, demonstrating functional vascularization. (**e,f**) 3D (upper) and transaxial (lower) PET images of a mouse injected with AB1-B/c-LUC cells (**f**) show ^18^F-FDG signals (white arrows) in the abdomen, whereas images of a control animal do not (**e**). The higher signal in 3D PET images is due to the bladder, through which ^18^F-FDG is excreted. Images are presented with the same scale and were corrected for injected dose and animal weight. SUV: Standardized Uptake Value.

Figure 7a,b show haematoxylin-eosin staining of explanted and paraformaldehyde-fixed tumors from AB1 cells. Although vascularization can be clearly observed only at the periphery of the masses, and their inner portions do not show identifiable vessels, there was no evidence of necrosis. Immunostaining with anti-CD31 antibody (which recognizes endothelial cells) showed that indeed a meshwork of capillaries is present inside the masses (Fig. 7c), providing sufficient vascularization to support tumor growth.

Contrast-Enhanced UltraSound (CEUS) scans of tumors growing *in vivo* show that the contrast medium initially distributes in the outer part of the masses and then slowly infiltrates the tumors (Fig. 7d and Supplementary Movie 1), indicating a slow blood flow in the inner part of the lesion.

These results were further confirmed by PET scans of mass-bearing mice, following i.v. injection of ^18^F-FDG. The detected signal, shown in Fig. 7e, indicates that the radioactive tracer has reached the mass through vascularization and that the mass has higher glucose consumption, a hallmark of tumors, than the surrounding abdominal organs^28^.

The results indicate that AB cells establish aggressive tumors that display features similar to human MM.

### Histopathological characterization of tumor masses generated by murine MM cell lines

We report the first IHC characterization of AB murine MM lines and tumors, since none was performed either on the original asbestos-generated lesions or on the AB cell lines derived from them^14^.

**Figure 8:**
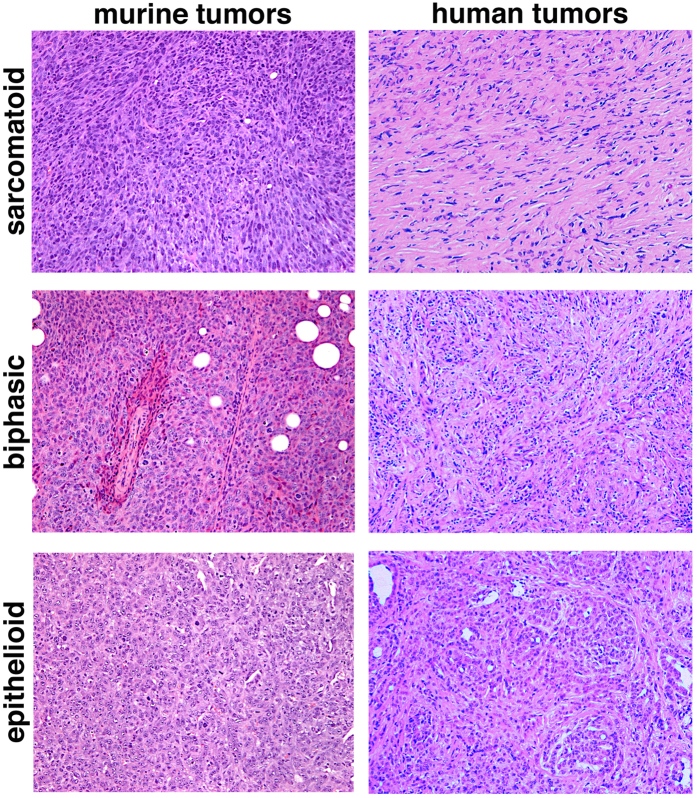
Murine and human tumors have similar morphologies. Sections of explanted tumor masses generated by injection of AB1, AB12 and AB22 cells in BALB/c mice were stained with hematoxylin-eosin (H&E), as were sections from human sarcomatoid, biphasic and epithelioid mesotheliomas. The architecture of murine tumors appears similar to that of the corresponding human masses.

**Figure 9:**
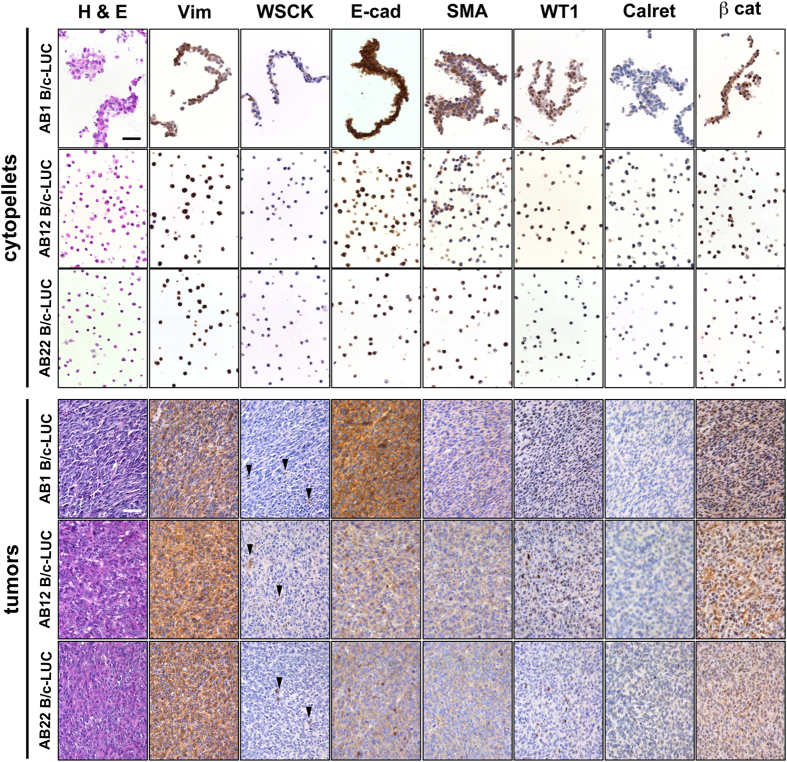
Immunohistochemical characterization of MM cell lines and tumors derived from them. Following detachment from culture dishes, cells were fixed and centrifuged; the pellets were then sectioned and stained with the indicated antibodies (cytopellet). Explanted tumor masses were fixed, sectioned and stained with the same antibodies as in cytopellets. Arrows indicate positive cells. All pictures were taken with the same magnification (10X). Bars in top left panels of H&E stain of cytopellets and tumors = 50 μm.

Explanted AB-derived sarcomatoid, biphasic and epithelioid tumors bear morphological similarities with samples of human biopsies of the same histological subtype (Fig. 8). However, when murine MM cells and the corresponding tumors were immunostained with antibodies routinely used for the diagnosis of human mesotheliomas, only vimentin yielded a positive signal on all cells and tumors, whereas only few AB1 cells were positive for wide spectrum cytokeratin (WSCK) and AB22 cells were positive for Wilm’s Tumor antigen (WT1). Both AB cells and tumors were positive for other epithelial markers, such as E-cadherin and β-catenin, and also yielded a signal for smooth muscle actin (SMA) (Fig. 9; Table 2 and Supplementary Table 2), whereas WSCK was expressed only in rare cells within all tumors. Interestingly, cytopellets of cultured cells and tumors derived from the same cell line display discrepancies in the expression of surface markers, as previously reported for human MM cell lines and tumors^27^. Both cells and tumor masses were overlayed with an irrelevant rabbit antibody, as a negative control, yielding no signals (Supplementary Figure 4).

IHC characterization of human MM is still ambiguous: a high variability of diagnostic markers has been observed due to reasons pertaining to the cells^29^ or to their manipulations^27^. Moreover, little consensus has been reached on which and how many markers should be used for a positive identification^3^,^30^. Our data support the notion that murine MM tumors express a variable set of histological markers.

### The response to pharmacological treatment of the murine MM syngeneic system

**Figure 10:**
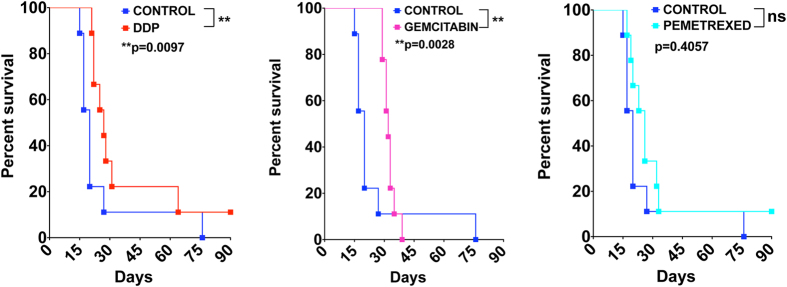
Tumors generated by murine MM AB1-B/c-LUC cells respond to pharmacological treatments used for human MM. Ten mice/group (control vs. treatment) were injected i.p. with 10^5^ AB1-BALB/c-LUC cells. The Kaplan-Meier curves show that the treatment with DDP (**a**) significantly extends the survival of MM-bearing mice. Gemcitabin (**b**) significantly delays the onset of the disease. Pemetrexed (**c**) does not significantly affect the survival.

In order to evaluate the response to pharmacological treatment of the syngeneic system, mice injected i.p. with AB1-B/c-LUC cells were treated with diamminedichloridoplatinum (DDP), Gemcitabin and Pemetrexed, as described in Materials and Methods, and their survival monitored. DDP and Gemcitabin treatments extended survival relative to the control group (Fig. 10), although in different ways. While DDP-treated mice survived longer than the control group throughout the observation period, more Gemcitabin-treated mice survived at early times, with little difference at later times (Fig. 10a,b). Pemetrexed treatment did not significantly extend survival (Fig. 10c). Although the multimodal administration of cisplatin (carboplatin)-pemetrexed extends survival of human patients^9^, the combined treatment is inefficient in a syngeneic mouse model^31^ and was not tested here.

Overall, the response of the syngeneic mouse MM system to pharmacological treatments is remarkably similar to the response of human MM^32^.

## Conclusion

In this report we describe a murine syngeneic system to study MM. The murine MM cells used here were obtained from tumors generated by the injection of asbestos fibers in the peritoneum of female BALB/c mice^14^ and were deemed *bona fide* mesothelioma cells. Our results show that AB cells exhibit phenotypical and functional features of MM *in vitro* and *in vivo*. In particular they express and secrete HMGB1, respond to it and produce tumors similar to human MM when injected in mice. Finally, tumors produced by AB1 cells respond to pharmacological treatments in a qualitatively similar way as human MM. Thus, we advocate the use of immuncompetent mice injected with AB cells (and in particular AB1 cells) as a preclinical experimental system for the study of MM development and treatment.

## Materials and Methods

### Cell lines, culture conditions and manipulations

Murine malignant mesothelioma (MM) AB1, AB12 and AB22 cells were obtained from Cell Bank Australia and cultured in RPMI 1640 (Life Technologies) supplemented with 5% (AB1 and AB12) or 10% (AB22) v/v fetal bovine serum (Life Technologies), 2 mM L-glutamine and 100 U/ml penicillin/streptomycin.

Each cell line was intraperitoneally (i.p.) injected in BALB/c mice to obtain tumors. The masses were explanted and mechanically disaggregated; the resulting cells were cultured as above and named AB1-B/c, AB12-B/c and AB22-B/c.

Luciferase-expressing cells were obtained by infecting the above cells with a 3^rd^ generation lentiviral vector carrying the luciferase gene (pLenti PGK V5-LUC Neo (w623-2); Addgene). Infected cells were selected with geneticin and maintained in culture as above. Cells generated from the original strains were named: AB1-LUC, AB12-LUC and AB22-LUC. Cells generated from the masses in BALB/c mice were named AB1-B/c-LUC, AB12-B/c-LUC and AB22-B/c-LUC.

### Mice

Animal experiments have been reviewed and approved by the Animal Care and Use Committees (IACUC) of both Ospedale S. Raffaele and Istituto di Ricerche Farmacologiche “Mario Negri”, which include “ad hoc” members for ethical issues. All experiments were performed in accordance with the approved guidelines. Animals were housed in the Institutes’ Animal Care Facilities, which meet international standards. Certified veterinarians who are responsible for health monitoring, animal welfare supervision, experimental protocols and procedures revision regularly checked them, in both institutions.

### qPCR

Total RNA from mouse primary mesothelial cells and from the original or manipulated murine MM cells was isolated using the Illustra RNAspin Mini kit (GE Healthcare) and retro-transcribed with oligo(dT) primers using a SuperScript II Reverse transcription kit (Invitrogen) following the manufacturer’s instructions. Quantitative real-time PCR, in a LightCycler480 (Roche) apparatus, was performed in triplicate with 5 μl of cDNA/sample and SYBR Green I master mix (Roche) using the following primers (final concentration 5 μM):

murine HMGB1 Fwd 5′-CCGGGAGGAGCACAAGAAGA-3′

murine HMGB1 Rev 5′-CCCTTTTTCGCTGCATCAGG-3′

murine β-actin Fwd 5′-AGACGGGGTCACCCACACTGTGCCCATCTA-3′

murine β-actin Rev 5′-CTAGAAGCACTTGCGGTGCACGATGGAGGG-3′.

Quantification was performed using the ΔCt method and β-actin gene was used for normalization.

### Western Blot and ELISA assay

Total cell extracts from primary murine mesothelial cells and original or manipulated murine MM cell lines were prepared using lysis buffer (Tris-HCl pH 7.4 20 mM, NaCl 150 mM, SDS 0.1%, Triton 1%, sodium deoxycholate 1%) and Protease Cocktail Inhibitor (Sigma-Aldrich). The total protein content was determined by Bio-Rad protein assay dye reagent (Bio-Rad). Twelve μg of proteins/sample were run in duplicate on a 12% SDS-PA gel and transferred to nitrocellulose membranes. The filters were blocked with 5% skim milk in Tris-buffered saline, pH 7.0, containing 0.1% Tween 20 (TBS-T) and probed with rabbit polyclonal anti-HMGB1 antibody (1:1000; Abcam) in TBS-T plus 5% milk overnight at 4 °C, washed several times with TBS-T, and incubated for 1h with rabbit anti-peroxidase antibodies (1:2000; Life Technologies) at RT. Western blots were visualized using the Western Blotting Luminol Reagent according to the manufacturer’s instructions (Santa Cruz). β-actin was revealed with a mouse monoclonal antibody (Sigma; 1:5000) and was used as loading control.

Quantification of the HMGB1 and β-actin bands was performed with the Fiji software (http://fiji.sc/Fiji).

The ELISA assay was carried out on the supernatant of cultured cells (indicated in Table 1) using an ELISA assay kit from IBL International (Germany) according to the manufacturer’s instructions and as described^10^.

### Migration and invasion assays

Migration and invasion assays were performed in Boyden chambers, seeding 5 × 10^4^ cells/chamber on the upper part of the filters. The medium was OPTIMEM in the upper chamber and OPTIMEM containing HMGB1 (30 ng/ml) in the lower chamber. After 3 hours, the filters were coloured with Giemsa stain (Sigma; 1:5 in H_2_O) and cells on the lower surface of the filter counted.

### Clear field microscopy

Cells in culture dishes were visualized with an Axio Observer Z1 microscope (Zeiss).

### Electron microscopy

Cells were grown on coverslips and fixed as monolayer in 2.5% glutaraldehyde in 0.1 M sodium cacodylate buffer pH 7.4 for 1 h at room temperature, washed three times with cacodylate buffer and fixed in 1% osmium tetroxide, 1.5% potassium ferrocyanide in 0.1 M cacodylate for 1h on ice. After several washes in distilled water samples were “en bloc” stained with 0.5% uranyl acetate in water overnight at 4 ^o^C. Samples were dehydrated in a graded ethanol series (30%, 50%, 70%, 80%, 99%, 96%; 5 minutes each and 3 washes with absolute ethanol, 10 min each). The samples were then infiltrated in a 1:1 ethanol/Epon 812 solution for 2 hours and in 100% Epon twice for 1h each. The coverslips were then layered on a drop of Epon and polymerized in an oven at 60 ^o^C for 48 hours. After separating the glass from the Epon block (by immersing it in liquid N_2_) a portion of the specimen was glued on top of an Epon block and mounted on a Leica Ultracut UCT ultramicrotome. Ultrathin (70–90 nm) sections were collected on copper grids and stained with uranyl acetate and Sato’s lead citrate before imaging with a ZEISS Leo AB 912 Omega transmission microscope. Images were acquired by a 2 k × 2 k bottom-mounted slowscan Proscan camera controlled by the EsivisionPro 3.2 software.

### Visualization of metaphase chromosomes

Metaphase spreads were visualized with a Nikon Eclipse 90i (Nikon Instruments) equipped with the acquisition and analysis Genikon software (Nikon Instruments) following overnight treatment of cells with colcemid (GibcoKaryoMAXColcemid, LifeTechnologies) solution in PBS (10 ng/mL) at 37 °C. Metaphases harvest was carried out according to standard protocols: briefly, trypsin detached cells were treated with hypotonic solution (0.075 M KCl for 15 min. at RT) and fixed in acetic acid/methanol (1:3 v/v). Air-dried metaphase spreads slides were analysed by QFQ banding following standard procedures; description of karyotypes and clonality criteria followed the International System for Human Cytogenetic Nomenclature recommendations (ISCN, 2013).

### Sequencing and bioinformatic analysis

Cell lines were sequenced on Illumina HiSeq 2500 High Output Mode using sequencing setting of single-end 51bp read long. Reads were aligned on reference genome (mm10) using bwa (v.0.5.10). In order to produce normalized Log2Ratio for each chromosome, we used SeqCNV (https://github.com/NKI-GCF/SeqCNV) provided by the Genomics core facility at NKI-Avi, which allows performing copy number analysis from low coverage sequence. Bash scripts from SeqCNV were used to generate read counts in non-overlapping genome windows of 20,000 bp and to correct the bin counts for the GC bias.

### *In vivo* BioLuminescence optical Imaging (BLI)

It was performed on mice after the i.p. injection of 7 × 10^4^ AB1-B/c-LUC cells using an IVIS SpectrumCT Preclinical *In Vivo* Imaging System (Perkin Elmer). The system is equipped with a low noise, back-thinned, back-illuminated CCD camera cooled at −90 ^o^C (quantum efficiency in the visible range above 85%). Before BLI each mouse received an intra-peritoneal injection of 6 g luciferin/kg body weight. During image acquisition, the animals were kept at 37^o^ C and under gaseous anesthesia (2–3% isoflurane and 1 lt/min O_2_). After luciferin injection dynamic BLI was performed from 0 to 30 minutes by acquiring an image every 2 minutes (exposure time = auto, binning = 8, f = 1 and a field of view equal to 13 cm (field C)) in order to detect the highest BLI signal. BLI image analysis was performed by measuring the total light flux (photons/seconds) in a Region of Interest (ROI) placed over the animal abdomen. Images were acquired and analyzed using Living Image 4.4 (Perkin Elmer).

### Ultrasound scans

Ultrasound (US) scans were carried out on anesthetized mice with a Vevo 2100 appartus (FUJIFILM VisualSonics Inc.) especially designed for the examination of small experimental animals. US images in B-mode (Brightness mode) were performed using a Vevo 2100 linear array transducer with a center frequency of 40 MHz (MicroScan MS 550D; 22–55 MHz; FUJIFILM VisualSonics Inc.).

Contrast Enhanced Ultrasound (CEUS) studies were performed using the MS250 linear transducer (fc = 21 MHz, 13–24 MHz; Vevo 2100; FUJIFILM, VisualSonics, Toronto, Canada) during intravenous bolus injection of Vevo MicroMarker (Bracco, Geneva, Switzerland) untargeted ultrasound contrast agent (CA), prepared following the instructions of the producer. A total volume of 50μl of MicroBubbles (MB) suspension (4.3 × 10^7^ MB/bolus) was injected in 4 seconds. Ultrasound data acquisition started immediately after CA injection. Imaging parameters were: transmit Power 4%; dynamic range 40 dB; center frequency 18MHz; frame rate 20 Hz; contrast gain 53 dB; gate 4; beam-width standard.

### PET scans

PET scans were performed using the small animal YAP-(S)-PET II tomograph (I.S.E. s.r.l., Italy)^28^ with an axial field of view (FOV) and a diameter of the transaxial FOV of 4 cm. The spatial resolution is 5.2 mm^3^ (FWHM, full width at half maximum) and the maximum of absolute sensitivity measured in the centre of FOV is 1.87% for the 50–850 KeV energy window.

[^18^F]FDG is routinely prepared at our institution for clinical use (European Pharmacopeia VIII ed.) with a radiochemical purity >99%. After a slight anesthesia with ether animals were injected in the tail vein with 7.60 ± 0.97 MBq of [^18^F]FDG in 50 μl of saline. Immediately before PET acquisition, mice were anesthetized with 2% isoflurane. PET scans started 60 min after tracer injection and lasted 30 minutes (six frames of 5 minutes each). PET data were acquired in list mode using the full axial acceptance angle of the scanner (3D mode) and then reconstructed with the expectation maximization (EM) algorithm^28^.

### Histopathology and Immunohistochemistry

For histopathological examination, samples were fixed in 10% neutral buffered formalin (NBF) for at least 24–48 hours, processed with a Tissue Processor Leica ASP300 S, and paraffin embedded (Embedding Center Leica EG1160). Sections of 4 μm were cut, stained with Haematoxylin-Eosin (H&E) and evaluated under a light microscope (Leica DM 2500). Representative images were captured with a digital camera (Leica DFC310 FX).

For immunohistochemistry 4 μm serial sections from each sample were immunostained with the primary antibodies listed in Supplementary Table 1 and incubated with appropriate biotinylated secondary antibody: goat anti-rabbit (VC-BA-1000-MM15, Vector Laboratories, USA). Sections were labelled by the avidin-biotin-peroxidase (ABC) procedure with a commercial immunoperoxidase kit (VECTASTAIN® Elite ABC-Peroxidase Kit Standard, VC-PK-6100-KI01, Vector Laboratories). The immunoreaction was visualized with 3,3′-diaminobenzidine substrate (Peroxidase DAB Substrate Kit, VC-SK-4100-KI01, Vector Laboratories) and sections were counterstained with Mayer’s haematoxylin. Negative immunohistochemical controls were prepared by replacing the primary antibody with an irrelevant one and known positive control sections were included in each immunolabeling assay.

### Pharmacological treatments

7 weeks old female BALB/c mice were obtained from Harlan Laboratories (Italy). They were injected i.p. with 10^5^ AB1-B/c-LUC murine mesothelioma cells. One week after inoculation mice were randomized into experimental groups and treatments started. All antitumor drugs were was administered intravenously (i.v.): Pemetrexed (Alimta, Eli Lilly), 200 mg/kg, every four days for four times (q4dx4); gemcitabine (Gembin, Actavis) 100 mg/kg, every four days for five times (q4dx5); cisplatin (DDP, Cisplatine, TEVA), 5 mg/kg, every seven days for three times (q7dx3). Control mice were treated with saline q4dx5. Mice were monitored daily and weighted at least twice a week throughout the experiments; they were sacrificed when severely distressed.

### Statistical analysis

Statistical analyses were performed with GraphPad Prism software, version 6.01 (GraphPad software, Inc., USA). Kaplan-Mayer survival curves were compared by the Gehan-Breslow-Wilcoxon test.

## Additional Information

**How to cite this article**: Mezzapelle, R. *et al.* Human malignant mesothelioma is recapitulated in immunocompetent BALB/c mice injected with murine AB cells. *Sci. Rep.* **6**, 22850; doi: 10.1038/srep22850 (2016).

## Supplementary Material

Supplementary Information

Supplementary Movie

## Figures and Tables

**Table 1:** HMGB1 is secreted by murine primary mesothelial and AB cells.

PMC	AB1	AB1-B/c-LUC	AB12	AB12-B/c-LUC	AB22	AB22-B/c-LUC
0.08	1.5	1.5	2.6	2.2	2.2	1.3

HMGB1 secreted over 16 hours was measured in the culture medium by ELISA. Values are expressed as ng of HMGB1 secreted by 10^6^ cells. PMC: Primary Mesothelial Cells.

**Table 2:** Immunohistochemical characterization of MM cells.

Primary Antibodies *(abbreviation)*	Cell culture			Tumors		
	AB1	AB12	AB22	AB1-B/c	AB12-B/c	AB22-B/c
Vimentin *(Vim)*	+	+	+	+	+	+
Wide Spectrum Cytokeratin *(WSCK)*	+/−	−	−	+/−	+/−	+/−
E-cadherin phosphor *(E-cad)*	+	+	+/−	+	+/−	+
Smooth Muscle Actin *(SMA)*	+	+^*^	+^*^	+/−	+/−	+/−
Wilm’s Tumor Antigen 1 *(WT1)*	−	?	+	−	−	−
Calretinin *(Calret)*	−	−	−	+/−	−	−
β-catenin (β*cat)*	+	+	+	+	+	+

(−) = absence of staining; (+/−) = weak staining; (+) = positive staining; (*) = some cells do not express the protein; (?) = uncertain.
