# Supplementary material for: Human malignant mesothelioma is recapitulated in immunocompetent BALB/c mice injected with murine AB cells
Source: Sci Rep. 2016 Mar 10;6:22850. doi: 10.1038/srep22850 (PMC4785401; doi:10.1038/srep22850)
Supplement: Supplementary Information [file srep22850-s1.pdf]

SUPPLEMENTARY MATERIAL

Human malignant mesothelioma is recapitulated in immunocompetent BALB/c mice injected with murine AB cells

Rosanna Mezzapelle[1], Eltjona Rrapaj[1,†], Elena Gatti[1], Chiara Ceriotti[1], Francesco De Marchis[1], Alessandro Preti[2], Antonello E. Spinelli[3,4], Laura Perani[3], Massimo Venturini[3,5], Silvia Valtorta[3,6], Rosa Maria Moresco[3,7], Lorenza Pecciarini[8], Claudio Doglioni[8,16], Michela Frenquelli[9], Luca Crippa[10], Camilla Recordati[11], Eugenio Scanziani[11,12], Hilda de Vries[13], Anton Berns[13], Roberta Frapolli[14], Renzo Boldorini[15], Maurizio D'Incalci[14] e Marco E. Bianchi[1,16,*] and Massimo P. Crippa[1,*]

[1]Chromatin Dynamics Unit, Division of Genetics and Cell Biology, San Raffaele Hospital, Milano, Italy;

[2]HMGBiotech, Milano, Italy;

[3]Experimental Imaging Center, San Raffaele Hospital, Milano, Italy;

[4]Medical Physics Unit, San Raffaele Hospital, Milano, Italy;

[5]Diagnostic Radiology Unit, San Raffaele Hospital, Milano, Italy;

[6]IBFM-CNR, Segrate, Italy;

[7]Health Sciences Dept., Milano Bicocca University, Milano, Italy;

[8]Pathological Anatomy Laboratory, San Raffaele Hospital, Milano, Italy;

[9]Molecular Oncology Unit, San Raffaele Hospital, Milano, Italy;

[10]ISTOVET, Besana in Brianza, Monza e Brianza, Italy;

[11]Fondazione Filarete, Milano, Italy;

[12]Università degli Studi, Milano, Italy;

[13]Division of Molecular Genetics, The Netherlands Cancer Institute, Amsterdam, The Netherlands;

[14]Mario Negri Institute, Milano, Italy;

[15]Division of Pathology “Maggiore Della Carità” Hospital, Novara, Italy;

[16]San Raffaele Vita-Salute University, Milano, Italy.

[†]current address; Unit of Pathology, Department of Health Sciences, University of Eastern Piedmont “Amedeo Avogadro”, Novara, Italy

[*]corresponding authors: crippa.massimo@hsr.it; bianchi.marco@hsr.it

SUPPLEMENTARY TABLE 1- Details of primary antibodies and procedures used for the immunohistochemical characterization of murine mesothelioma cell lines and masses used in this study.

Antigen	Primary antibody	Source	Antigen retrieval	Working dilution	Incubation time	Detection system
Vimentin	EPR3776 Rb mon[a]	Epitomics	HIER[c]	1:500	1h RT[d]	ABC[e]
Cytokeratin Wide Spectrum	Z0622 Rb poly	Dako	Digestion (Pepsin)	1:600	1h RT[d]	ABC[e]
E-cadherin Phospho	EP913(2)Y Rb mon[a]	Epitomics	HIER[c]	1:600	1h RT[d]	ABC[e]
Smooth Muscle Actin (SMA)	#1184-1 Rb mon[a]	Epitomics	HIER[c]	1:150	1h RT[d]	EnVision[f]
Calretinin	PA5-34688 Rb poly[b]	Thermo Scientific	HIER[c]	1:300	1h RT[d]	ABC[e]
Wilm's Tumor Antigen 1 (WT1)	CAN-R9(IHC)-56-2 Rb mon[a]	Epitomics	HIER[c]	1:100	1h RT[d]	ABC[e]
β-catenin	6B3 Rb mon[a]	Cell Signaling	HIER[c]	1:150	1,5 h RT[d]	ABC[e]

[a]Rabbit monoclonal; [b]Rabbit polyclonal; [c]heat-induced epitope retrieval, pH=9.0; [d]one hour at room temperature; [e]Avidin-biotin complex (Vectastain Elite ABC kit PK-6100, Vector Laboratories Inc., Burlingame, CA, USA); [f]Dako EnVision™ System (DakoCytomation, Glostrup, Denmark).

SUPPLEMENTARY TABLE 2 – Grading of the immunohistochemical staining and semiquantitative evaluation of the samples.

GRADING

GRADE	EXTENT	INTENSITY
0	0	0
1	1-25%	weak
2	25-50%	moderate
3	50-75%	strong
4	>75%	

SEMIQUANTITATIVE EVALUATION: = EXTENT × INTENSITY

**Primary Antibodies** *(abbreviation)*	**Cell culture**			**Tumors**		
	**AB1**	**AB12**	**AB22**	**AB1-B/c**	**AB12-B/c**	**AB22-B/c**
**Vimentin** *(Vim)*	8(4×2)	12(4×3)	12(4×3)	8(4×2)	8(4×2)	12(4×3)
**WideSpectrum Cytokeratin** *(WSCK)*	1(1×1)	0(0×0)	0(0×0)	1(1×1)	1(1×1)	1(1×1)
**E-cadherin phospho** *(E-cad)*	12(4×3) M+C	8(4×2) M+C	3(3×1) C	12(4×3) M+C	3(3×1) C	8(4×2) M+C
**Smooth Muscle Actin** *(SMA)*	8(4×2)	3(3×1)	6(3×2)	3(3×1)	3(3×1)	3(3×1)
**Wilm's Tumor Antigen 1** *(WT1)*	0(0×0)	6(2×3)?	9(3×3)	0(0×0)	0(0×0)	0(0×0)
**Calretinin**						

(Calret)	0(0×0)	0(0×0)	0(0×0)	1(1×1)	0(0×0)	0(0×0)
**β-catenin** *(βcat)*	8(4×2) N+C	12(4×3) N	8(4×2) N	6(3×2) N(C)	8(4×2) N(C)	2(2×1) N(C)

M = membrane; C = cytoplasm; N = nucleus.

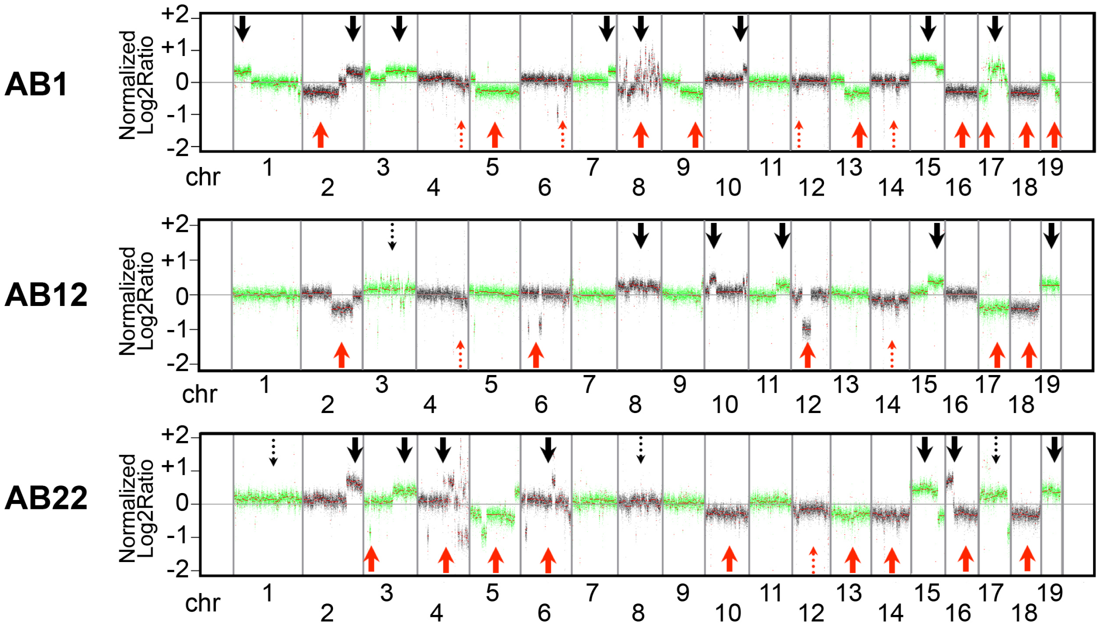

Supplementary Figure 1 – Partial chromosomal imbalances in MM cell lines detected by next-generation sequencing – Copy number variations (CNVs) in AB cell lines. Low coverage sequence data show broad intra- and interchromosomal heterogeneity. Log2Ratio values are normalized to the entire genomic sequence. Deletions are indicated by red arrows and duplications by black ones; bold arrows indicate CNVs involving large chromosomal regions, and dashed arrows indicate CNVs involving small chromosomal regions.

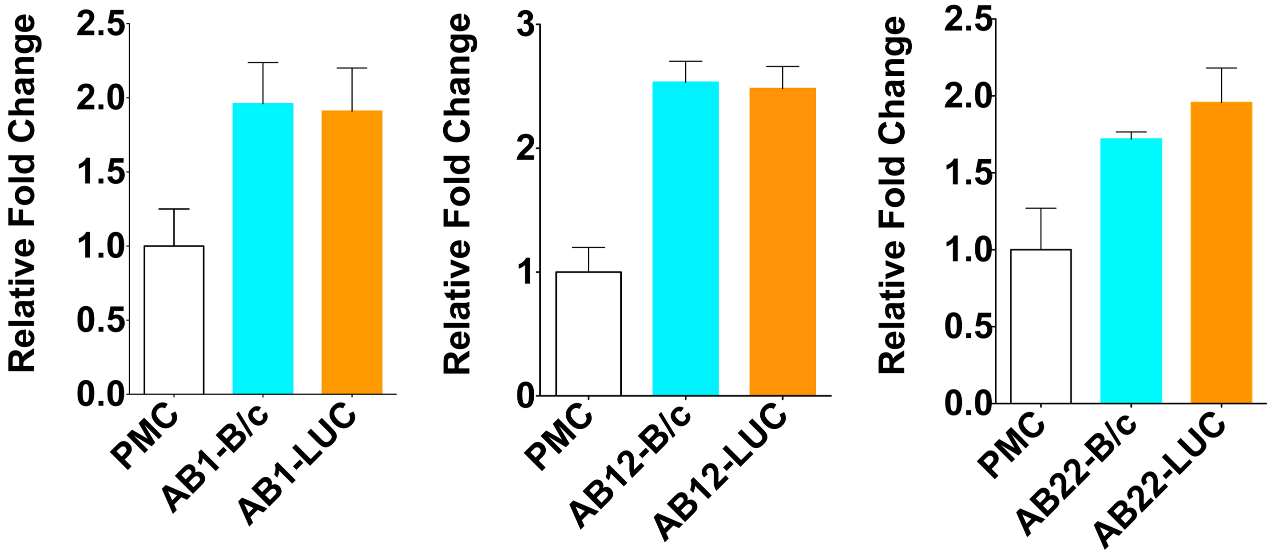

Supplementary Figure 2 - High levels of HMGB1 mRNA expression in manipulated murine MM cell lines – Total RNA from primary mesothelial cells or the indicated cell lines was extracted, retrotranscribed and amplified with the appropriate primers, as described in Materials and Methods. Also in these cell lines the endogenous HMGB1 mRNA is expressed about twofold higher in MM cell lines that in primary mesothelial cells (compare with Figure 3a).

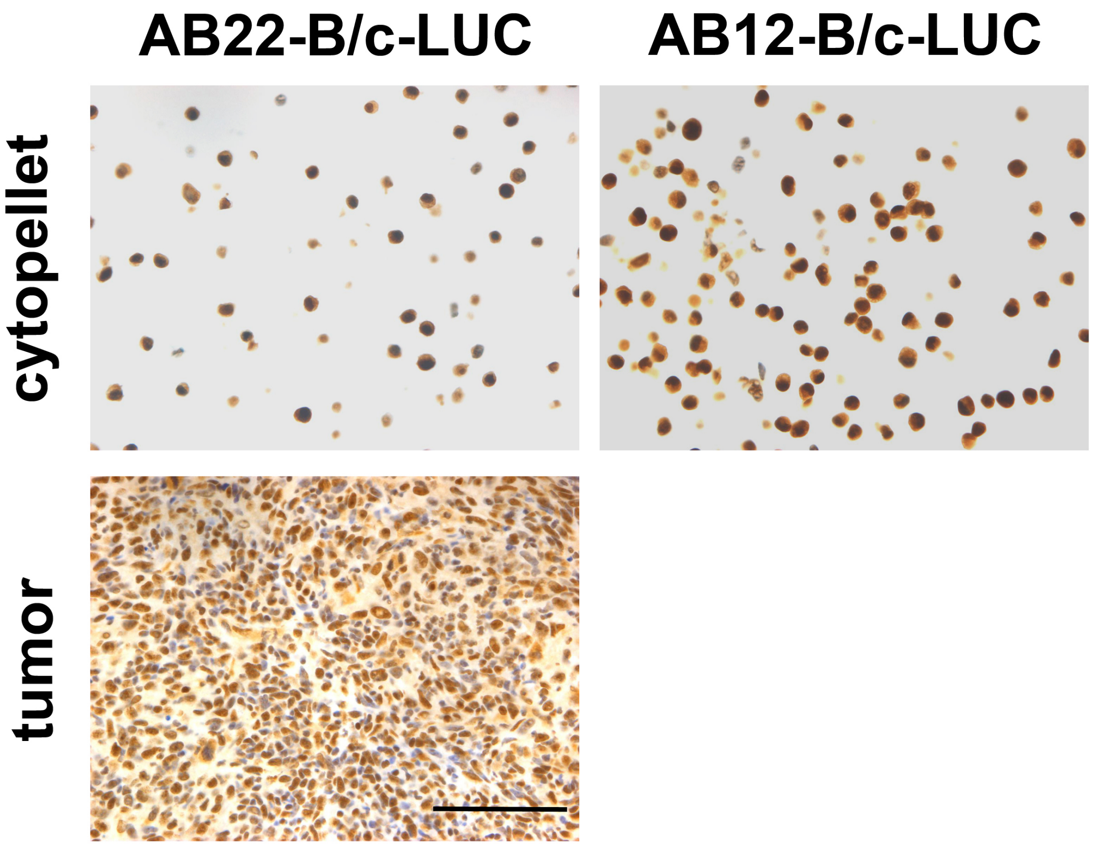

Supplementary Figure 3 – HMGB1 localizes to the nucleus and the cytoplasm of AB12-B/c-LUC cells and in AB22-B/c-LUC cells and derived tumors. – Immunostaining with anti-HMGB1 antibodies show the nuclear and cytoplasmic localization of HMGB1 both in cultured cells and tumor tissue. Bar in bottom panel = 100 μm

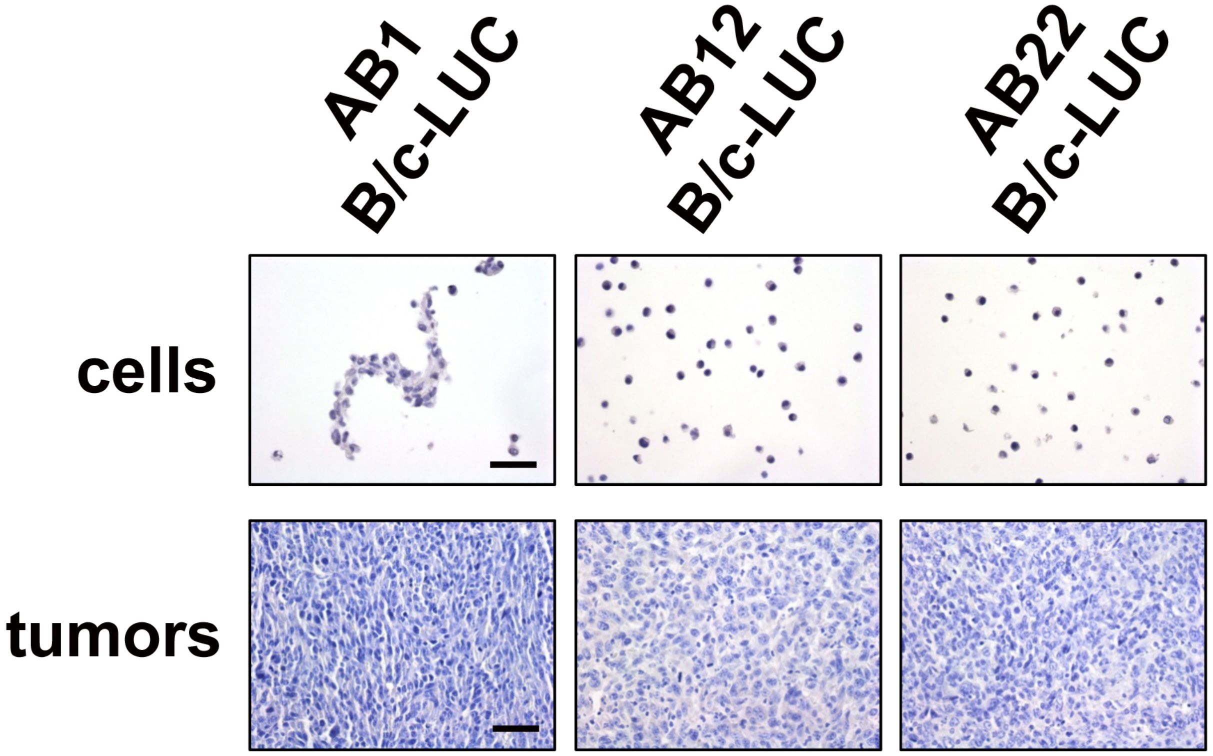

Supplementary Figure 4 – Immunohistochemical negative controls of MM cell lines and tumors derived thereof. - Cells detached from culture dishes were fixed, pelleted and sectioned, while explanted tumor masses were fixed and sectioned. All sections were stained with an irrelevant rabbit polyclonal antibody. All pictures were taken with the same magnification (10X). Bars in left panels = 50 μm.

LEGEND TO SUPPLEMENTARY MOVIE

Supplementary Movie 1 - Contrast-Enhanced UltraSound (CEUS) movie, obtained as described in Materials and Methods, showing that the intravenously delivered contrast bolus infiltrates tumor masses.
